# Talent Research in Sport 1990–2018: A Scoping Review

**DOI:** 10.3389/fpsyg.2020.607710

**Published:** 2020-11-25

**Authors:** Joseph Baker, Stuart Wilson, Kathryn Johnston, Nima Dehghansai, Aaron Koenigsberg, Steven de Vegt, Nick Wattie

**Affiliations:** ^1^Kinesiology and Health Science, York University, Toronto, ON, Canada; ^2^School of Human Kinetics, University of Ottawa, Ottawa, ON, Canada; ^3^Department of Human Movement Sciences, University of Groningen, Groningen, Netherlands; ^4^Faculty of Health Sciences, Ontario Tech University, Oshawa, ON, Canada

**Keywords:** expertise, athlete, development, giftedness, selection

## Abstract

Several recent systematic and targeted reviews have highlighted limitations in our understanding of talent in sport. However, a comprehensive profile of where the scientific research has focused would help identify gaps in current knowledge. Our goal in this scoping review was (a) to better understand what others have done in the field of research (e.g., what groups have been examined using what research designs and in what areas), (b) to summarize the constituent areas of research in a meaningful way, (c) to help identify gaps in the research, and (d) to encourage future research to address these gaps. Peer-reviewed articles written in English that met several inclusion criteria were analyzed. A total of 1,899 articles were identified, and the descriptive findings revealed a relatively narrow focus of research on talent in sport. Specifically, the majority of examined articles focused on (a) males only, (b) the sport of soccer, (c) perceptual cognitive variables, (d) developing athletes, (e) adult samples, and (f) cross-sectional designs. For better or worse, the concept of talent remains a central element of how coaches, practitioners, and scientists think about athlete development. Findings from this scoping review highlight the continued need to explore issues related to talent identification, selection, and development in more diverse samples (e.g., female athletes and younger ages) and contexts (e.g., from Africa, Asia, and South America). There is also a clear necessity to focus on under-researched areas using alternative methodologies.

## Introduction

Scientific study of exceptional individuals can be traced to the mid-1800's and the work of Francis Galton. Among the samples he considered in his work were wrestlers and rowers (oarsmen), making this the first known study of talent in sport (Galton, 1869). While Galton showed strong family clustering for eminence in several other areas of achievement, he indicated that the data for sport were less conclusive. Since this initial foray, public and scientific discourse on the notion of talent and innate ability has increased considerably. In addition to this interest among the scientific community to understand the predictors of exceptional achievement, strong global interest in sport as a source of revenue (e.g., in the case of professional sports; Heitner, 2019) and/or political capital (e.g., for nations to succeed at the international competitions such as the Olympics; Houlihan and Green, 2008) has created additional incentive for national policies on athlete development and talent identification (e.g., Canada's “Sport for Life' Model; Higgs et al., 2019). The notion of talent is central to many models and policies relating to athlete development, as reflected in the practice of identifying and selecting talented athletes at early ages, in order for them to develop in the most beneficial environments. These practices presuppose that talent is an innate characteristic, something that exists and can be identified early in an athlete's career, and that if identified, will predict later success and expertise (Howe et al., 1998; Baker et al., 2018).

The assumption that early identification and selection will lead to more positive athlete development outcomes and subsequently better sport outcomes makes talent identification, selection, and development critical areas of inquiry for researchers and sport practitioners. Perhaps more notably, ineffective or inaccurate decisions have important repercussions for all stakeholders involved (e.g., dropout, decreased motivation, misplaced resources, and investment). Despite the increased attention, however, research suggests that the ability to recognize, capture, and develop talent is imperfect at best (Baker and Wattie, 2018). However, for better or worse, the concept of talent remains a central element of how coaches, practitioners, and scientists think about athlete development.

Several recent reviews (see below) have highlighted the limitations in our knowledge regarding talent in sport (Baker and Wattie, 2018). This limited understanding is reflected in the inconsistent definitions and operationalizations of talent, which have led to a wide range of methodologies used (Baker and Wattie, 2018; Baker et al., 2019; Collins et al., 2019), low predictive validity in talent selections (Koz et al., 2012), and poor quality of existing evidence (Johnston et al., 2018). Ultimately, these inconsistencies and gaps in knowledge trickle down to practitioners attempting to make evidence-informed decisions. In the absence of relevant, specific knowledge, gaps may be filled with untested assumptions and inaccurate information.

Given the lack of research in many areas of talent science (e.g., accuracy of early talent decisions, Johnston et al., 2018), high-quality scientific research is needed in order to (a) determine the reliability and validity of talent identification and selection initiatives, (b) inform evidence-based models of athlete development, and (c) identify gaps in current understanding and directions for future work. This scoping review focuses on the final element, with the goal of providing a comprehensive overview of the current body of evidence for talent in sport to better understand where researchers have focused their attention and to indicate gaps requiring further attention. This objective is especially suited to a scoping review. Previous systematic reviews have explored talent in sport generally (e.g., Issurin, 2017), soccer specifically (Bergkamp et al., 2019), and used longitudinal methods to explore the topic of talent by comparing skilled and less skilled athletes (Johnston et al., 2018). Collectively, these authors have noted the lack of a strong evidentiary foundation for understanding talent in sport and/or the implications of this absence for explaining the processes and limitations of human potential. These reviews (among others) are important contributions to the literature. For example, the recent review by Sarmento et al. (2018) provides an excellent synthesis of talent identification and development in male soccer. Similarly, Johnston et al. (2018) examined longitudinal and retrospective studies from 1990 to 2015 and provided descriptive trends for that sub-sample of articles. While such specificity in systematic reviews and meta-analyses is a necessity for detailed syntheses of research findings, this approach cannot provide a comprehensive understanding of the broader context of talent research. An understanding of the “landscape” of the literature to date would be helpful for researchers and practitioners to substantiate and emphasize where gaps in knowledge are present. If we use the analogy of a forest to represent research on talent in sport, at present we do not know how large that forest is, its shape, in what ways is it growing, or, importantly, what species (i.e., sports) are present. A scoping review of talent research is valuable for understanding where overgeneralizations, as well as over-/underestimates of knowledge exist.

Broadly, this review aims to (a) better understand what others have done in the field of research (e.g., what groups have been examined using what research designs and in what areas), (b) summarize the constituent areas of research in a meaningful way, (c) help identify gaps in the research, and (d) encourage future research to address these gaps. Specifically, this review endeavors to illuminate the types and frequencies of study designs (i.e., the methodological approaches), the areas of focus (relative age, physiology, genetics, etc.), and the samples examined (age, size of sample, sport, sex, competition level, and location) for a broad range of studies on talent and talent related research.

## Methods

### Scoping Review

The aim of a scoping review is to aggregate available information on a specific topic by exploring the existing literature to better understand current trends and identify gaps. Scoping reviews are particularly useful for understanding complex and/or diverse issues in an area and are commonly used to include literature with a range of different study designs and methods (Davis et al., 2009; Peters et al., 2015). Scoping reviews typically precede systematic reviews because they identify and summarize the key characteristics/parameters worth consideration (Sucharew and Macaluso, 2019). In this case, a range of systematic reviews have proceeded, examining specific questions related to talent identification with no understanding of the literature as a whole, making a scoping review the ideal next step in addressing this gap (Sucharew and Macaluso, 2019).

### PRISMA-ScR and Inclusion Criteria

A broad but customized search was completed to identify relevant studies of talent in sport according to the Preferred Reporting Items for Systematic Reviews and Meta-Analyses extension for Scoping Reviews (PRISMA-ScR) statement guidelines (Moher et al., 2009; Tricco et al., 2018). Although the intention was to include as broad a profile of talent research as possible, some restrictions were made. Studies were excluded from the final review for the following reasons:

1. Non-sport focus: for example, studies of talent in music, physical education, and dance.
2. Non-athlete focus: studies of coach or referee expertise and/or development.
3. Not empirical studies: all non-data-driven studies (i.e., reviews, position statements, instrument design, and methodological papers).
4. Non-peer-reviewed studies: theses, dissertations, conference abstracts, and other non-peer-reviewed outputs (e.g., commentaries).
5. Non-English studies: due to limited language capabilities of the research team, only research articles written in English were included in the analyses.

### Key Search Terms and Search Strategy

In accordance with the PRISMA-ScR guidelines, the search strategy for identifying articles was broken down into two phases. Phase 1 consisted of searching three electronic databases—Web of Science, PsychInfo, and SPORTDiscus—in the time period of January 1990–December 2018^1^. Studies were identified using the following search terms to search in the title and abstract of the articles identified: “talent AND sport,” “expertise AND sport,” “giftedness AND sport,” “expert performance” AND “sport,” “elite performance” AND “sport,” and “talent” AND “athletes.” In order to determine the search terms, the researchers completed a preliminary-scoping review to identify the common language used in the literature when exploring “talent” in sport. This allowed for a relatively broad search without losing focus for the aims of this review. Phase 2 consisted of a secondary search of external sources such as the reference list of articles found in phase 1 and reference lists in books and book chapters. On completion of these phases, the study's author(s), title, and year of publication were recorded, and articles were sorted to eliminate duplicates. From the list of unique entries, the publication's title was read to discern whether the article was written in English and was in the form of a complete, peer-reviewed journal study. From this refined list, a more intensive assessment took place, which required obtaining the abstracts and the full-text articles.

### Groupings

Each article in this refined list was evaluated by an independent assessor for the following attributes: publication year, participants' sex (grouped as male, female, mixed), participants' age [grouped as child (age 3–5), youth (age 6–11), adolescent (age 12–17), adult (age 18+)^2^], participants' nationality, sample size (<20 participants, 20–50 participants, 51–100 participants, 101–200 participants, 201–500 participants, >500 participants), and sport. In addition, the skill level of participants in each study was grouped according to three categories: *beginner, developing*, and *expert*. Any unskilled participants (such as novices in expert vs. novice paradigms) were considered *beginners*, while *expert* participants were determined using the criteria noted by Baker et al. (2015) and Swann et al. (2015). Any samples not categorized as beginner or expert (e.g., athletes recruited to a talent development program or those at levels of skill lower than expert) were categorized as developing. Further, study design was categorized as cross-sectional (e.g., Buxens et al., 2011; Roca et al., 2014), intervention/short tracking (e.g., Tallir et al., 2005; Foskett et al., 2009), longitudinal/prospective (Lidor et al., 2007; Vink et al., 2015), retrospective (i.e., any study tracking historical patterns such as studies of athletes' time spent in deliberate practice, Baker et al., 2003; Young and Salmela, 2010), or some combination of these categories (e.g., a combined retrospective and longitudinal design, Güllich and Emrich, 2014). Finally, efforts were made to categorize the focus of each study relative to the following broad categories: anthropometric characteristics, biomechanical–technical skills, developmental pathways, physiological characteristics, perceptual–cognitive characteristics, psychological characteristics, relative age effects, training/practice, and other^3^.

### Reliability of Coding

Coding of each study was performed by all members of the authorship team. To ensure the reliability of coding for each study, sub-samples of 200 randomly selected studies were evaluated by two or three independent reviewers at the start of the coding process. Any disagreements in coding were discussed and clarified among the research team to ensure coding was consistent. Once all articles were coded, one researcher reviewed and amalgamated the work of all researchers, ensuring consistency of coding terminology and checking a further 200 randomly selected studies for coding accuracy.

A descriptive analysis of all studies meeting our inclusion criteria is presented below. In addition, we were especially interested in the characteristics of research among studies focusing on male and female athletes exclusively and, to this end, we examined age, sample size, skill level, and study design in each of these groups separately.

## Results

**Figure 1:**
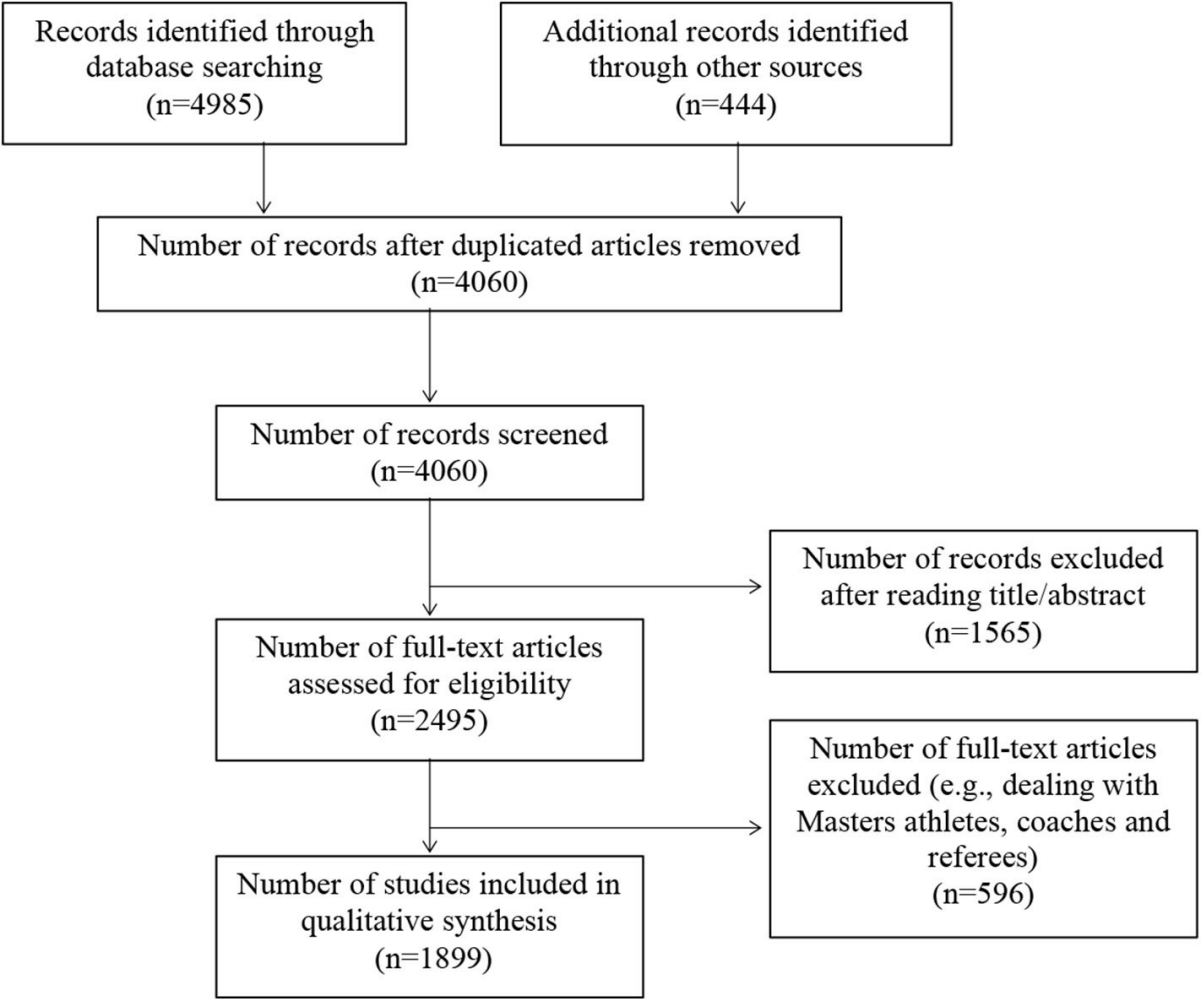
PRISMA flow chart showing number of records collected and number of eligible records after the screening process.

**Figure 2:**
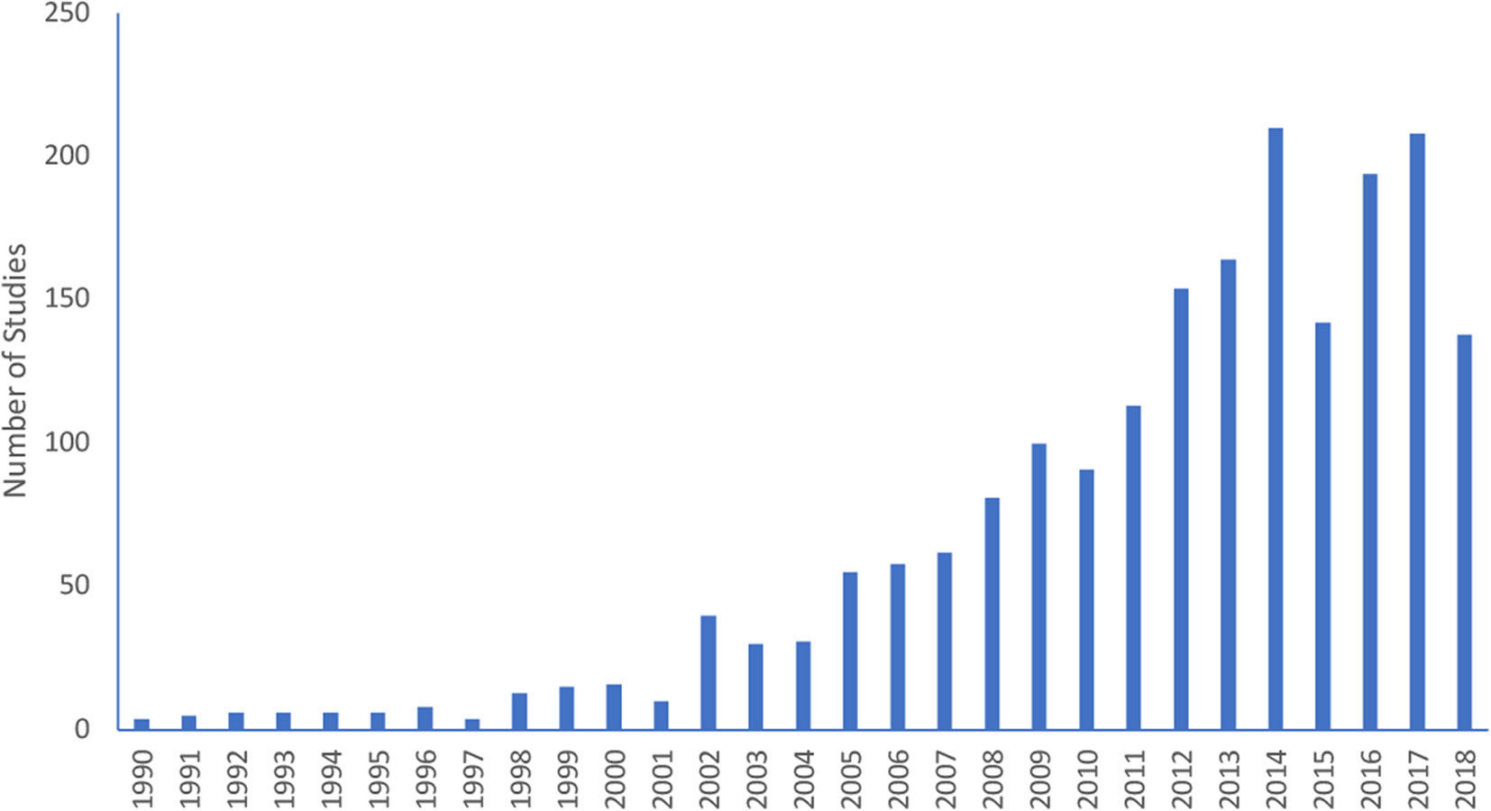
Number of studies by publication year.

The initial search identified 4,060 articles, of which 1,899 met the inclusion criteria and were considered for analyses (see Figure 1). Articles with multiple experiments/studies that included different samples were considered separately but treated as one article in the overall profile. Figure 2 illustrates the profile of study publication dates, strongly reflecting the growing interest in this area of research.^4^ Descriptive data for sex, age, sample size, skill level, and study design are presented in Table 1. Similarly, descriptive results for country and sport are presented in Table 2. A summary of the research foci explored in the total list of studies is presented in Table 3 followed by male- and female-specific results.

**Table 1:** Descriptive statistics for sex, age, sample size, skill level, and study design for the overall sample, males, and females.

	**Overall**	**Males**	**Females**
	***N*** **(%)**	***N*** **(%)**	***N*** **(%)**
**Sex**			
Female	203 (10.3%)	–	–
Male	863 (43.8%)	–	–
Mixed	612 (31.1%)	–	–
Not reported	292 (14.8%)	–	–
**Age**			
Childhood	0 (0%)	–	–
Youth	64 (3.2%)	28 (3.2%)	7 (3.4%)
Adolescent	356 (18.1%)	155 (18.0%)	37 (18.2%)
Adult	823 (41.8%)	390 (45.2%)	66 (32.5%)
Mixed child and youth	3 (0.2%)	0 (0%)	2 (1%)
Mixed child, youth, and adolescent	3 (0.2%)	2 (0.2%)	1 (0.5%)
Mixed child, youth, adolescent and adult	8 (0.4%)	4 (0.5%)	1 (0.5%)
Mixed youth and adolescent	123 (6.2%)	72 (8.3%)	10 (4.9%)
Mixed youth, adolescent, and adult	52 (2.6%)	5 (0.6%)	10 (4.9%)
Mixed adolescent and adult	374 (19.0%)	145 (16.8%)	53 (26.1%)
Not reported	164 (8.3%)	62 (7.2%)	16 (7.9%)
Any child^a^	14 (0.6%)	6 (0.6%)	4 (1.4%)
Any youth^b^	253 (10.4%)	113 (10.6%)	33 (11.7%)
Any adolescent^c^	916 (37.5%)	388 (36.3%)	114 (40.6%)
Any adult^d^	1258 (51.5%)	561 (52.5%)	130 (46.3%)
**Sample Size**			
<20	413 (21.0%)	181 (21.0%)	58 (28.6%)
20–50	577 (29.3%)	266 (30.8%)	64 (31.5%)
51–100	290 (14.7%)	119 (13.8%)	29 (14.3%)
101–200	209 (10.6%)	101 (11.7%)	23 (11.3%)
201–500	176 (8.9%)	74 (8.6%)	13 (6.4%)
501+	270 (13.7%)	111 (12.9%)	12 (5.9%)
Not Reported	35 (1.8%)	11 (1.3%)	4 (2.0%)
**Skill Level**			
Beginner	53 (2.7%)	13 (1.5%)	0 (0%)
Developing	858 (43.6%)	414 (48.0%)	83 (40.9%)
Expert	307 (15.6%)	136 (15.8%)	36 (17.7%)
Mix of beginner and developing	223 (11.3%)	99 (11.5%)	15 (7.4%)
Mix of beginner, developing, and expert	92 (4.7%)	31 (3.6%)	11 (5.4%)
Mix of beginner and expert	66 (3.4%)	22 (2.5%)	12 (5.9%)
Mix of developing and expert	325 (16.5%)	138 (16.0%)	39 (19.2%)
Not reported	46 (2.3%)	10 (1.2%)	7 (3.4%)
**Study Design**			
Cross-sectional	1344 (68.2%)	592 (68.6%)	147 (72.4%)
Intervention/short-tracking	178 (9.0%)	87 (10.1%)	15 (7.4%)
Longitudinal	145 (7.4%)	71 (8.2%)	14 (6.9%)
Mixed cross-sectional/intervention	2 (0.1%)	1 (0.1%)	0 (0%)
Mixed cross-sectional/longitudinal	4 (0.2%)	2 (0.2%)	0 (0%)
Mixed cross-sectional/retrospective	19 (1.0%)	7 (0.8%)	1 (0.1%)
Mixed short-tracking/retrospective	2 (0.1%)	0 (0%)	0 (0%)
Retrospective	276 (14.0%)	103 (11.9%)	26 (12.8%)

a *Includes all studies with any children among the participants*.

b *Includes all studies with any youth among the participants*.

c *Includes all studies with any adolescents among the participants*.

d *Includes all studies with any adults among the participants*.

**Table 2:** Most popular sports and countries.

**Sport^a^**	***N* (%)**
Soccer	442 (22.4%)
Basketball	102 (5.2%)
Tennis	81 (4.1%)
Handball	81(4.1%)
Rugby	80 (4.1%)
Combat sports^b^	74 (3.8%)
Volleyball	72 (3.7%)
Golf	62 (3.1%)
Ice hockey	53 (2.7%)
Australian rules football	52 (2.6%)
Gymnastics^c^	51 (2.6%)
Swimming	38 (1.9%)
Cricket	38 (1.9%)
Triathlon	31 (1.6%)
Athletics—general^d,e^	29 (1.5%)
Baseball	27 (1.4%)
Badminton	27 (1.4%)
Field hockey	19 (1.0%)
Athletics—long distance^d,f^	18 (0.9%)
Table tennis	17 (0.9%)
Rowing	14 (0.7%)
Shooting	14 (0.7%)
Canoe–Kayak	13 (0.7%)
Cycling^g^	13 (0.7%)
Sailing	11 (0.6%)
Mixed	302 (15.3%)
Not Reported	46 (2.3%)
**Country^a^**	
Australia	173 (8.8%)
United Kingdom^h^	172 (8.8%)
Germany	116 (5.9%)
USA	94 (4.8%)
Canada	85 (4.3%)
France	79 (4.0%)
Spain	77 (3.9%)
Portugal	58 (2.9%)
Netherlands	56 (2.8%)
Belgium	49 (2.5%)
Italy	36 (1.8%)
Brazil	29 (1.5%)
Switzerland	28 (1.4%)
Poland	23 (1.2%)
China	22 (1.1%)
South Africa	22 (1.1%)
Japan	20 (1.0%)
Israel	15 (0.8%)
Sweden	14 (0.7%)
New Zealand	13 (0.7%)
Finland	12 (0.6%)
Ireland	10 (0.5%)
Mixed	152 (7.7%)
Not Reported	437 (22.2%)

a *Only sports and countries with at least 10 studies were included*.

b *Combat sports included studies of boxing, fencing, judo, karate, kendo, kickboxing, krav maga, martial arts (general), taekwondo, and wrestling*.

c *Gymnastics included all disciplines such as artistic gymnastics, rhythmic gymnastics, and trampoline*.

d *Note that there were other categories for Athletics—Middle Distance, Athletics—Throws, Athletics—Jumps, and Athletics—Sprint that did not reach the threshold of n = 10 studies to be included in this table. All studies that focused on an aspect of Athletics or Track and Field are included together n = 69*.

e *Athletics—General included any study that could not be placed in one of the specific athletics categories*.

f *Athletics—long distance included any study that focused on runners in what the International Association of Athletics Federations would consider a distance event (e.g., 10,000 m and marathon)*.

g *Cycling included road, mountain, BMX, etc*.

h *United Kingdom included all studies where participants were noted as coming from the UK as well as from England, Scotland, Wales, and Northern Ireland*.

**Table 3:** Top categories identified in talent analysis for overall, male-only, and female-only samples.

**Keywords**	**# of Studies**^**a**^		
	**Overall**	**Male-Only**	**Female-Only**
	***N*** **(%)**	***N*** **(%)**	***N*** **(%)**
Perceptual cognitive skills	727 (25.5%)	332 (25.5%)	72 (25.6%)
Physiological characteristics	518 (18.2%)	263 (20.2%)	62 (22.1%)
Psychological characteristics	300 (10.5%)	103 (7.9%)	25 (8.9%)
Anthropometric characteristics	279 (9.8%)	145 (11.2%)	37 (13.2%)
Relative age effects	204 (7.2%)	100 (7.7%)	14 (5.0%)
Training/practice	192 (6.7%)	87 (6.7%)	15 (5.3%)
Developmental pathways	216 (7.6%)	78 (6.0%)	19 (6.8%)
Biomechanical/technical skills	311 (10.9%)	145 (11.2%)	32 (11.4%)
Other^b^	102 (3.6%)	47 (3.6%)	5 (1.8%)

a *Total category entries exceed total studies because many studies were assigned to multiple categories*.

b *Other includes studies on genetics, birthplace/community size effects, and family influences as well as other topics that were not classifiable using the above categories*.

### Sex

The majority of studies (43.8%) focused on male-only participants with just 10.3% examining female-only samples. Just over 31% of studies in the review included mixed samples of males and females. Surprisingly, nearly 14.8% of studies did not explicitly report the sex of their participants, although in most cases, authors likely believed sex was implied through the populations under investigation (e.g., professional teams or samples associated with athlete development “academies”).

### Age

Most talent research investigated adult samples (41.8%), followed by mixed samples of adults and adolescents (19.0%), and adolescents only (18.1%). A significant proportion of research (28.6%) used mixed samples (i.e., two or more age categories). Very few studies examined children or youth and ~8% did not report the age groups in their study.

### Sample Size

Sample sizes ranged from single participant studies (e.g., Jones, 2006) to nearly half a million participants (i.e., 4,742,321 participants in Del Campo et al., 2010). The largest proportion of studies were in the smaller sample size categories, that is, the 20–50 category (29.3%) and the <20 category (21.0%). At the other end of the size range, a meaningful proportion of studies (13.7%) had samples larger than 500 participants.

### Participant Nationality

**Figure 3:**
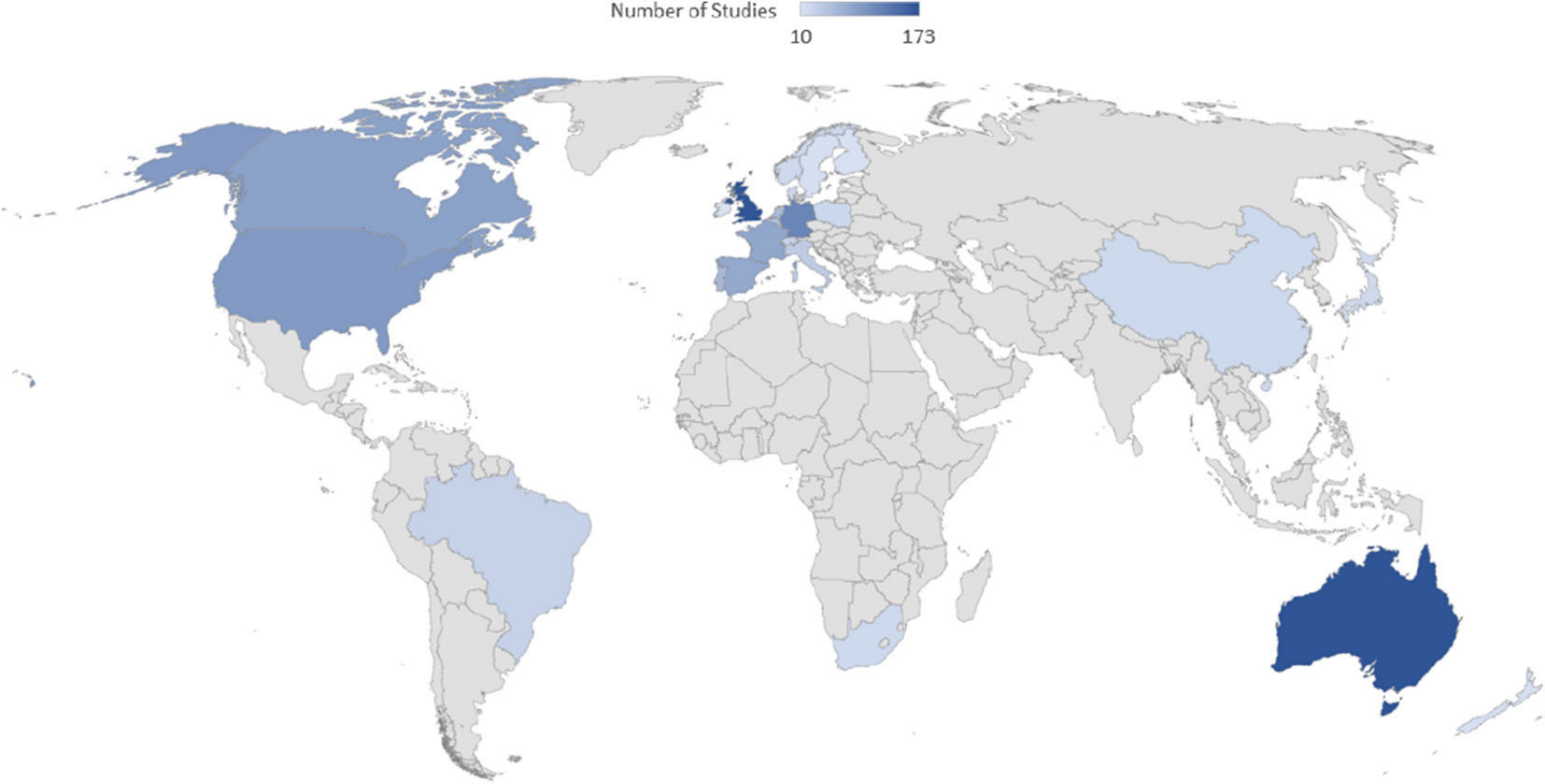
Distribution of athlete samples from around the world. Darker shaded areas denote a greater number of studies.

Athletes from 54 countries were represented among the reviewed studies. The country with the greatest number of studies conducted upon its athletes was Australia (*n* = 173 studies or ~9% of all studies) followed closely by the United Kingdom with 172 studies (8.8%) and Germany with 116 studies (~6%).^5^ As noted in Table 1, there are 23 countries with >10 studies focusing on athletes from these nations. The international distribution of these samples is illustrated in Figure 3. Approximately 8% of studies included mixed country samples and 22% of studies did not report the nationality of their participants.

### Sport

Seventy-five sports were represented in the studies reviewed (see Table 2). The largest group of studies involved soccer players (*n* = 442 studies) comprising ~22% of the overall sample. The next largest was basketball, with 102 studies (5%) along with tennis, handball, rugby, and combat sports with 81, 81, 80, and 74 studies, respectively.

### Skill Level

For the skill level comparison, the greatest number of studies examined participants who were “developing” (43.6%), followed by expert-only (15.6%), and beginner-only (~3%). Approximately 36% of studies used mixed groups, which is not surprising given the dominance of comparison-based designs. Among the mixed groups, the largest group was a mix of developing and expert athletes (16.5%), followed by beginner and developing athletes (11.3%). Surprisingly, given the perceived dominance of the expert vs. novice paradigm in expertise research (Abernethy et al., 1993; Baker et al., 2015), only 3.4% of research reviewed included beginners compared with experts according to our classifications.

### Study Design and Research Foci

The vast majority of research in this area (68.2%) utilized cross-sectional research designs (see Table 1). The next most common approach was retrospective designs (14.0%) followed by intervention/short-tracking, and longitudinal designs, which made up 9.0 and 7.4% of the sample, respectively. The remaining 2.6% of studies utilized combinations of the above designs (see Table 1).

Analysis of the research topic resulted in 2,845 individual text codes, which were grouped into nine general themes (see Table 3). The largest category of studies was for examinations of perceptual–cognitive factors (*n* = 727 studies, 25.5%), followed by physiological characteristics (*n* = 518 studies, 18.2%), psychological characteristics (*n* = 300 studies, 10.5%), and anthropometrics (*n* = 279 studies, 9.8%).

### Sub-Analysis: Male vs. Females

Comparisons of studies with male- and female-only samples (Table 1) suggested very similar profiles across the groups for the study designs used (i.e., predominately cross-sectional for both groups). However, there was some evidence that, compared with males-only studies, females-only studies more often utilized a mix of groups, including adolescents and adults, with fewer adult-only samples. Males-only studies also reflected a larger proportion of studies with mixed youth and adolescent samples compared to the females-only group. There were also notable differences in sample sizes with a greater proportion of females-only studies using smaller samples (i.e., <20 participants) and a lower proportion of studies with large samples (i.e., 501+) compared to males-only studies. Further, there was a greater proportion of males-only studies that focused on developing athletes compared to females-only studies (i.e., 48 vs. 41%, respectively). Finally, although there were some differences between males and females in the research topics examined (e.g., greater percentage of studies for males than females), these differences were generally small, suggesting a similar profile of research exploration in both groups.

## Discussion

Having a broad understanding of gaps in our understanding of sporting talent is essential for making evidence-based and knowledge-informed decisions. Our goal in this descriptive analysis was to identify these gaps. Results highlight the considerable scope and depth of research done by scientists in fields ranging from physiology and biomechanics, to developmental and sport psychology. Moreover, there was evidence of considerable growth in this field over the last 30 years. However, despite the broad range of samples and topics used in prior research, there was clear evidence of imbalances in where research efforts have been focused.

The descriptive data indicate a large focus on perceptual–cognitive research, although this may be due to our search terms. For example, our search may have missed studies in fields such as physiology and biomechanics because these fields are less likely to use words like talent, expertise, and giftedness. In particular, the rapid growth of the scientific field of expertise, which is strongly rooted in psychology, might explain the dominance of perceptual–cognitive work in our analysis. That said, the large amount of perceptual–cognitive work being done highlights the considerable evidence available in this area. The large number of studies in this area (and others such as physiological and psychological characteristics) suggests some potential value in further, more targeted systematic reviews or meta-analyses.

Outside of, or in combination with, the traditional expertise discipline on cognitive psychology, this scoping review suggests a range of other opportunities for future work. In particular, researchers are encouraged to devote greater attention to female athletes since the constraints and developmental models may differ from their male counterparts (e.g., females physically mature earlier and operate in a system with fewer financial incentives and less support, Handelsman, 2017; Curran et al., 2019). The historical discrepancy of funding for female sports could explain the limited available research; however, with the growth of female sports, it is imperative to better understand factors related to female-specific talent development.

Further, nearly a quarter of studies in this area focused on soccer. While this suggests soccer may be better suited for an evidence-based understanding of talent, the applicability of non-sport-specific research to a specific sport (i.e., soccer research to basketball) may be limited given the unique developmental constraints associated with each sport and the domain specificity of perceptual–cognitive skills and expertise attainment (Loffing et al., 2012). Moreover, asymmetries in the sports studied may negatively affect our ability to accurately parse the sport-specific influences of talent (i.e., innate qualities) from those that result from domain-specific practice. As proposed by Baker et al. (2019), talent may emerge through the interaction of person, environment and task, making the characteristics of how it presents in a given domain potentially specific to that situation. While the volume of research on soccer highlights the global significance of the sport and its associated funding, more research in other sports can parse out similarities and differences across sports to better understand the concept of talent.

A similar concern relates to research conducted in different countries, such as the relevance of research exploring the development of German athletes (e.g., Güllich and Emrich, 2014) to athletes in other countries, particularly those with fundamentally different athlete development systems (e.g., Kenya or Jamaica). While some of the developmental factors and relationships may be common (e.g., the value of early diversification and later deliberate practice as advocated in models such as the Developmental Model of Sport Participation has been found in some instances to apply across countries and sports; see Côté and Vierimaa, 2014), there is also the potential that the unique athlete development systems found in different countries may affect the relevance of these results outside of the context in which they were measured. Greater research attention is needed to explore the generalizability of much of this research across contexts. In particular, the results of the current review (see Table 2) highlight the under-representation of research from countries within South America, Asia, and Africa. Certainly, the volume of research from these regions is not reflective of the socio-cultural popularity of sport or the sporting talent in these regions. This finding may reflect a smaller volume of research emanating from those regions and/or our exclusion of non-English language publications from our review (see limitations below). Either way, it is very likely our understanding of talent identification and development largely reflects the systems and sports from Europe, North America, and Australia (see Henrich et al., 2010). One measure to navigate through language or funding barriers is increasing international collaborations between research teams from various regions. For example, it may be worth pursuing a large-scale international project that purposefully includes research and practitioners from around the world to document diversity in (1) definitions and concepts of talent, (2) evidence for and against talent, and (3) different applied talent identification and development models. Such global connections can have a wide range of benefits including added knowledge in the field in the form of theoretical contributions, and empirical data from cross-regional comparisons of athletes and sports.

The vast majority of research examined utilized cross-sectional designs with adult samples. As noted elsewhere (Johnston et al., 2018), the lack of longitudinal studies in talent science is problematic, but perhaps not surprising given the logistical and administrative costs of this type of research. All the same, the last decade has seen an increasing focus on work of a longitudinal nature (e.g., Elferink-Gemser et al., 2007; Till et al., 2013; Schorer et al., 2017; Williams et al., 2020). This is critical given that talent, in its essence, is a time-constrained variable. One could argue that it is not possible to infer or evaluate talent with a cross-sectional approach or from looking retrospectively with adult samples. Furthermore, given the importance of sport participation toward achieving the health benefits associated with the recommended daily physical activity levels (Kjønniksen et al., 2009; Janssen and LeBlanc, 2010), and the considerable resources dedicated to identifying and developing talent in sport, it might be necessary to consider ways to facilitate the longitudinal tracking of athletes. For example, a national registry, which assigns unique identifiers to each athlete independent of individual sport organizations, would provide a means to track athlete participation and progression across a sport or multiple sports. This type of comprehensive tracking system could also expand the potential questions and methods that could be explored in talent research. For example, Baker et al. (2019) proposed talent as innate, multi-dimensional, emergenic, dynamic, and symbiotic. A registry for longitudinal tracking, supplemented with something akin to a multidisciplinary “talent census,” would allow us to test the veracity of these proposed elements/definitions of talent, as well as others (e.g., Howe et al., 1998). A talent census could also positively contribute to talent transfer initiatives (Rea and Lavallee, 2017), as well as a better understanding of constraints on talent–environment relationships (i.e., the dynamic and symbiotic features of innate talent).

There were also some differences between the samples of males-only studies and females-only studies, although what this variation means for the quality of evidence for males vs. females is debatable. Research with males utilized larger sample sizes than research with females, which may ultimately affect the stability and longevity of the study results since research designs with small samples may have been statistically underpowered. Notably, there were no sex differences in the types of study designs used, as both male- and female-based studies predominantly employed cross-sectional approaches. Understandably, the cost of longitudinal research may impede some researchers in conducting optimally designed studies; however, if one of the goals of talent research is to identify key factors contributing to athlete development, when long-term tracking is not feasible or possible, cross-sectional approaches should consider more diverse groups.

It is noteworthy that only ~36% of the studies included any comparison groups featuring any combination of beginner, developing, and elite athletes. Similarly, most of the studies examined adult athletes only, and again, very few studies compared athletes of different age-cohort groups. This suggests, beyond retrospective study designs, that there is a lack of lifespan developmental data on talent in sport. Given that sport organizations are required to make selections relatively early in athletes' careers (e.g., to select athletes for “representative” teams), it would be vital to examine key performance- and development-related variables to determine how they change over time and how they relate to future attainment. The current, predominantly, adult-only focus does little to shed light on these relationships and may mislead those developing policies for long-term athlete development by suggesting a greater evidence base than actually exists. Similarly, without detailed developmental data, it is difficult for research to adequately inform practitioners regarding how to identify talent. There may also be a need to consider the taxonomy and nomenclature used within the talent literature, specifically concerning how athlete samples are described and categorized. An anecdotal observation and concern that arose during the coding of data was the description of some samples as “elite youth athletes” or “talented athletes,” and that the skill level of some youth samples could be overinflated in the literature. For example, in some countries and specific sports, participating in at the college/university level constitutes the highest, most elite level of participation for that developmental stage. However, this is not true of all countries and sports, and even differs within countries. As such, one challenge is to navigate a type of relativism that exists in classifying athletes (at any age) as “elite.” Given cultural and contextual differences, there may be a benefit to more explicitly justifying and validating the classification/description of developing athletes.

Based on our observations, very few studies explored variation among elite level athletes. The implicit notion that “development stops” at the elite level is problematic, and it limits our understanding of talent and expertise development. Indeed, with the exception of the theory of deliberate practice and Australia's Foundations, Talent, Elite, Mastery (FTEM) model, there is little information about how athletes continue to develop once they reach elite levels, and what factors distinguish “expertise” from “eminence” (see Baker et al., 2015). However, differentiating athletes at the same level of participation is challenging. Outcomes such as number of games played, or years played, can be confounded by age (see Collins et al., 2016). Matching participants based on age can help to address this limitation (see Güllich et al., 2017) but brings the added challenge of potentially small sample sizes. Small samples are a reality of studying talent (Baker and Wattie, 2018), which forces us to confront some research conventions. As Ploutz-Snyder et al. (2014, p. 1251) suggest, in “scientifically amazing settings,” we may need to challenge the precept that only “big-n” studies are worthwhile. As such, when considering athletes in the highest echelons of expertise, it may be necessary to appraise our criteria of statistical significance, power, effect size metrics, and “acceptable” levels of tolerances for type I and II error rates (Bacchetti, 2010; Bacchetti et al., 2011; Abt et al., 2020).

Furthermore, it was surprising to see the large number of studies that did not report key descriptive variables. For instance, nearly 15% did not clearly report the sex of the athletes in their study, and while the sex may have been obvious to those knowledgeable about the sport (e.g., Baker et al., 2005; Deprez et al., 2015), if this information was not explicitly stated, it was deemed “not reported.” A similar result was found for the country the samples were drawn from, where 22% did not clearly report this information (e.g., Farrow and Abernethy, 2002; Bishop et al., 2014). Again, it may have been possible to infer this information from the authors of the studies, but it is often inappropriate to do so given the international makeup of many research teams and the propensity for researchers to move or work across countries and institutions during their careers. Researchers are encouraged to diligently report this information in future work as these data are necessary for larger analytical approaches (e.g., systematic reviews and meta-analysis) for the development of sound, evidence-based policy.

## Limitations

While this review provides insight into gaps in our knowledge of talent in sport, there were some limitations to our approach. An important limitation that future reviews should consider is collating the definition of talent used in the reviewed studies. While this would require tremendous resources, it would greatly contribute to our understanding of a wide range of definitions of talent and how this is conceptualized in research. Furthermore, due to the large number of studies in this review and our objectives, we were not able to assess the quality of the papers reviewed. It would have been valuable to determine whether the same studies that did not explicitly mention their sample's sex were the same that did not mention the country of the athletes, suggesting that a proportion of low-quality studies are having an overall influence on the profile of work in this area. Finally, our review was restricted to English-speaking articles only and, therefore, any talent research in other languages was missed. More generally, the exclusion of work from non-English-speaking researchers is a significant limitation to our understanding of talent, athlete development, and sport expertise; incorporating work from other regions of the world in different languages may contribute to a more comprehensive understanding of “talent.”

## Concluding Thoughts

To conclude, this scoping review emphasizes the imbalance in our sources of information and understanding of sporting talent. Generally, research in this field is overrepresented by relatively small samples of male adults, using cross-sectional designs of developing soccer players. This profile of research suggests several important areas for future work in order to better understand the complexity of sporting talent. Future research would benefit from identifying longitudinal variables to track in a wide range of sports considering participants from across competitive levels (preferably comparing amateur to elite) and sexes.

## Data Availability Statement

The original contributions presented in the study are included in the article/supplementary material, further inquiries can be directed to the corresponding author/s.

## Author Contributions

All authors contributed to the creation of this manuscript, involved in the extensive article review, and reviewed versions of the final manuscript prior to submission.

## Conflict of Interest

The authors declare that the research was conducted in the absence of any commercial or financial relationships that could be construed as a potential conflict of interest.

## Footnotes

^1^This timeframe was chosen to ensure the majority of articles published in that year would have made it into these search engines/databases to allow for tracking growth in this area over time.

^2^Note: Studies of Masters athletes were not included.

^3^These categories were generated through a simple text-based analysis of study keywords and article titles, and reflect the most common foci identified. A study could have multiple areas of focus.

^4^The low number of studies for 2019–2020 is undoubtedly due to more recent studies not being reflected in current search databases.

^5^Note: If we include studies identifying participants from England with those specifying the United Kingdom, this grouping would make up 7% of all studies (second only to Australia). Designation of England or United Kingdom was based on the information provided in the authors' original study.
